# Indications of unicompartmental knee arthroplasty and high tibial osteotomy would be different to achieve successful long-term outcome

**DOI:** 10.1186/s43019-025-00296-z

**Published:** 2025-10-28

**Authors:** Kang-Il Kim, Yeonseo Kim, Jun-Ho Kim

**Affiliations:** 1 Department of Orthopaedic Surgery, Center for Joint Diseases, Kyung Hee University Hospital at Gangdong, 892 Dongnam-ro, Gangdong-gu, Seoul, 134-727 Republic of Korea; 2 Department of Orthopaedic Surgery, School of Medicine, Kyung Hee University, Seoul, Korea; 3 Department of Orthopaedic Surgery, Hallym Sacred Heart University Hospital, Hallym University, 22, Gwanpyeong-ro 170beon-gil, Dongan-gu, Anyang-si, 13496 Gyeonggi-do Korea

**Keywords:** Unicompartmental knee arthroplasty, Medial open-wedge high tibial osteotomy, Long-term, Indication, Osteoarthritis

## Abstract

**Background:**

Although both unicompartmental knee arthroplasty (UKA) and medial open-wedge high tibial osteotomy (MOWHTO) are widely accepted surgical options for medial compartment osteoarthritis, there is limited evidence from long-term outcomes to confirm and refine their established indications. This study aimed to evaluate the long-term clinical and radiologic outcomes of UKA and MOWHTO when performed according to their established indications at a single institution, and to characterize the demographic and preoperative radiographic differences associated with surgical selection.

**Methods:**

Patients who underwent UKA or MOWHTO for medial compartmental OA with a minimum 10-year follow-up were retrospectively reviewed. Preoperative characteristics, including age and the degree of medial OA using Kellgren-Lawrence grading, clinical outcomes, and radiologic parameters, including hip-knee-ankle angle (HKAA), medial proximal tibial angle (MPTA), and OA progression in the patellofemoral compartment, were compared. Survivorship based on the conversion to total knee arthroplasty was also evaluated.

**Results:**

The current study included 79 UKAs and 140 MOWHTOs with a mean 13.2 ± 1.7 years follow-up. Preoperatively, the UKA group had significantly older age (*P* < 0.001) and more advanced degree of medial OA (*P* < 0.001) than the MOWHTO group. Postoperative clinical outcomes were not significantly different between the groups. Radiologically, the UKA group had significantly less varus alignment and larger MPTA than the MOWHTO group (all,* P* < 0.001). Although the proportion of OA progression in the patellofemoral joint was higher in the MOWHTO group than in the UKA group at the latest follow-up (*P* = 0.012), there was no significant difference in anterior knee pain. At the mean 13-year follow-up, survival rates were not significantly different between the UKA (96.2%) and MOWHTO (98.6%) groups.

**Conclusions:**

Both UKA and MOWHTO demonstrated excellent long-term outcomes when performed under their established indications for medial compartment OA. Patients selected for UKA were older, had more advanced OA, less varus alignment, and a larger MPTA compared with those undergoing MOWHTO, consistent with published selection criteria. Radiographic progression of patellofemoral arthritis occurred more frequently after MOWHTO than after UKA, although this finding was not associated with clinical significance.

## Background

In the treatment of medial compartmental osteoarthritis (OA), both unicompartmental knee arthroplasty (UKA) and medial open-wedge high tibial osteotomy (MOWHTO) are widely accepted surgical options that can delay or avoid conversion to total knee arthroplasty (TKA) in selected patients [1–3]. Recent expert consensus and systematic reviews have clearly delineated their respective indications, providing evidence-based criteria for patient selection [4–7].

In general, MOWHTO is indicated for younger, active patients with varus malalignment and mild-to-moderate medial OA as a joint-preserving procedure, whereas UKA is reserved for relatively older patients with advanced anteromedial OA and preserved alignment, functioning as a partial joint replacement [4–7]. Although some overlap in indications is inevitable, consensus statements emphasize that each procedure should be applied to distinct patient populations, and using these criteria is critical to achieve durable outcomes [4–9].

While several comparative studies have reported satisfactory long-term outcomes for both UKA and MOWHTO [2, 3], many included patients with overlapping indications, limiting interpretation of results. Consequently, there remains a paucity of evidence regarding long-term outcomes when each procedure is performed according to its established indications.

The present study aimed (1) to report the long-term clinical outcomes, radiologic findings, and survivorship of UKA and MOWHTO when performed under their established indications [6, 7, 10–15] at a single institution, and (2) to characterize the demographic and preoperative radiographic differences between groups to assess whether patient selection corresponded to established indications. We hypothesized that both procedures would demonstrate satisfactory long-term outcomes when performed according to established indications [6, 7, 10–15], and that patient demographics and radiographic characteristics would differ between groups in a manner consistent with previously reported selection criteria.

## Methods

### Patients and study design

A retrospective cohort study identified 271 consecutive knees that underwent 95 medial UKAs and 176 MOWHTOs procedures for medial compartmental knee OA at a single institution between February 2008 and July 2013. Approval was obtained from the institutional review board prior to the study (KHNMC2023-09–027). At our institution, we performed medial UKA and MOWHTO with the following indications and a consistent philosophy over the study period.

Although these operations are indicated for medial compartmental OA, UKA is a kind of joint replacement surgery with the opening the knee joint whereas HOWHTO is a joint preserving, lower limb surgery without joint operation. Therefore, we differentiated the indication for both surgeries supported by prior literature and the above concept. Surgical indications for UKA and MOWHTO were based on previously published consensus statements [6, 7, 10–16]. Medial UKA was indicated for symptomatic patients with anteromedial OA as follows [7, 10–14]: (1) age ≥ 60 years and Kellgren–Lawrence (K–L) grade ≥ 3 or age < 60 years and K–L grade 4 [14, 17], (2) functional cruciate and collateral ligaments [10, 14], (3) maximum 10° of a correctable varus deformity [14], and (4) asymptomatic lateral and patellofemoral compartmental pathology [13]. Meanwhile, MOWHTO was indicated for symptomatic patients with medial compartmental OA as follows: [6, 15, 16, 18] (1) age ≤ 70 years, (2) K–L grade 2 and 3, (3) isolation for mechanical axis realignment of tibial origin (medial proximal tibial angle [MPTA]:87° ± 3°) for varus malalignment ≥ 5°, and (4) body mass index (BMI) < 35 kg/m^2^0.

In cases with overlapping indications, the final decision between UKA and MOWHTO was made through a structured discussion between the patient and surgeon, including surgical concepts, expected outcomes, patient’s activity, and postoperative rehabilitation, within the framework of established clinical criteria.

The inclusion criteria for the current study were patients who had either UKA or MOWHTO with a minimum follow-up period of 10 years. The exclusion criteria for UKA and MOWHTO included patellofemoral OA of K–L grade > 3, lateral OA of K–L grade > 2, secondary OA including inflammatory arthritis, hemophilic arthropathy, post-traumatic OA, combined ligamentous instability or previous reconstruction surgery, range of motion(ROM) < 100°, > 20° of flexion-contracture (FC), and revision cases [15, 19].

**Fig. 1:**
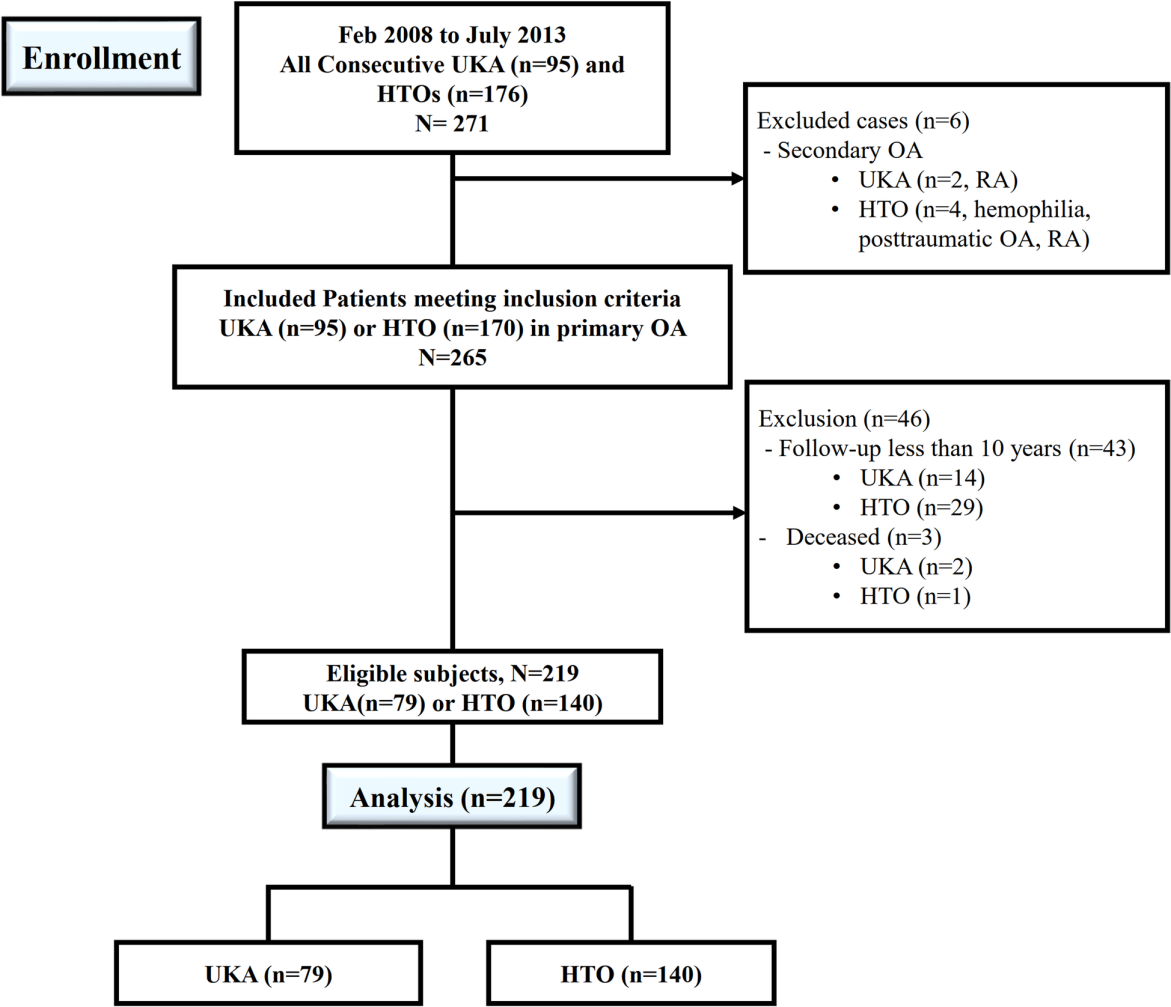
Flow diagram illustrating patient enrollment. Overall, 219 (80.8%) were enrolled in the current study. UKA, unicompartmental knee arthroplasty; HTO, high tibial osteotomy; OA, osteoarthritis; RA, rheumatoid arthritis

Of 271 knees, 219 knees were finally enrolled and analyzed in the current study, including 79 UKAs and 140 MOWHTOs (Fig. 1). The mean follow-up was 13.2 years (range, 10–16.4 years). Of the 219 knees, postoperative clinical evaluations were available for 214 knees, excluding 5 knees with survivorship failure. Postoperative radiologic evaluations were available for 204 knees, excluding 10 knees from the telephone survey for clinical outcomes.

### Surgical procedures and rehabilitation protocols

All the surgical procedures were performed by a single surgeon. In the UKA group, cemented Miller-Galante fixed-bearing (FB)(Zimmer-Biomet, Warsaw, IN, USA) or Oxford phase III mobile-bearing (MB)(Zimmer-Biomet; Warsaw, IN, USA) was performed using a standard technique [20, 21]. The selection of the FB or MB technique was based on the study period; FB-UKA was performed from February 2008 to August 2010 (*n* = 19) and MB-UK was performed from October 2010 to July 2013. Biplanar MOWHTO was performed with a minimally invasive technique and fixed using a medial locked-plate system (TomoFix; Synthes, Switzerland) [22–25]. The target of correction was significantly different as UKA aimed to achieve neutral or slightly varus alignment in terms of resurfacing arthroplasty. On the other hand, MOWHTO required valgus alignment aiming for the mechanical axis near or slightly below a point 62.5% of the tibial plateau from the medial edge, according to the arthroscopic cartilage status [21–23, 26, 27].

Both groups underwent similar rehabilitation protocols but not the same. No splint braces were required in all cases. On the day after the surgery, passive and active ROM, quadriceps setting, and ankle pump exercises were performed. Partial weight-bearing ambulation with crutches was initiated when pain was tolerable in all patients. Patients were permitted to begin full weight bearing without crutches at postoperative 6 weeks for MOWHTO patients while UKA patients started full weight bearing at postoperative 4 weeks.

### Evaluation

The clinical outcomes included ROM including FC and further-flexion (FF), American-Knee-Society(AKS) score [28], and Western-Ontario and McMaster-Universities-Osteoarthritis-Index (WOMAC) [29]. These clinical outcomes were assessed preoperatively and at the latest follow-up and compared between the two groups. Minimal clinically important difference (MCID) values were adopted to assess the clinically significant differences between the groups [30]. The MCID value was set based on previous studies [31–35] to compare the proportion of patients who achieved clinically meaningful improvements between the UKA and MOWHTO groups [36]. Moreover, anterior knee pain was assessed as a binary variable (presence or absence) based on patient-reported symptoms during clinical follow-up. Finally, the presence of anterior knee pain at the latest follow-up was evaluated for patellofemoral OA.

Radiological outcomes included the degree of medial compartment OA using the K–L grading system [37] on a standing anteroposterior(AP) view, HKAA(hip-knee-ankle angle) [38], MPTA [39], and mechanical lateral distal femoral angle(mLDFA) [40]. Moreover, the degree of patellofemoral OA was assessed using the K–L grading system in the Merchant view, and the incidence of radiologic progression of patellofemoral OA was analyzed in the two groups.

Survivorship failure (defined as the conversion to TKA) and mode of failure (defined as the cause of conversion to TKA) of both procedures were evaluated and determined by chart review. Survival rates were compared between the two groups. Furthermore, postoperative complications including surgical site infection, wound dehiscence, fixation failure, bearing dislocation, and revision surgery were recorded.

For the subgroup analysis, long-term clinical outcomes were compared in patients with overlapping indications between the UKA and MOWHTO groups. Overlapping indications were defined as varus malalignment between 5° and 10°, anteromedial OA of K–L grade 3 with age between 60 and 70 years, or anteromedial OA of K–L grade 4 with age < 60 years.

### Statistical analysis

A post hoc power analysis using significance set at an alpha of 0.05 was performed to determine whether the sample size had sufficient power to detect significant differences. All variables that were significantly different had > 80% power, which demonstrated that the sample size of the current study was adequately powered [41].

Continuous variables were analyzed using the Student *t*-test, and categorical variables were analyzed using chi-squared or Fisher’s exact tests. Preoperative and postoperative clinical and radiological variables were compared using the paired *t*-test. Kaplan–Meier survival analyses were performed with conversion to TKA and reoperation as the endpoint, and log-rank tests were performed on the survival curves, which showed intergroup differences in survival rates between two groups. To determine the reliability and reproducibility of the outcomes, all measurements were performed by two authors (J.-H. K. and Y.K.). Data were analyzed using SPSS software (version 23.0; IBM Corp., IL, USA), R statistical software (version 4.0.2), and G-power (version 3.1).

## Results

Patients in the UKA group (60.6 ± 5.1) were significantly older than those in the MOWHTO group (54.4 ± 8.6, *P* < 0.001) and other preoperative parameters were comparable (Table 1).

**Table 1:** Patients’ demographic data^α^

	UKA	MOWHTO	*P* value
Number of knees	79	140	
Number of patients	75	113	
Sex, male/female	9/66	20/93	0.137
Age, years	60.6 ± 5.1	54.4 ± 8.6	< 0.001
BMI, kg/m^2^	27.2 ± 2.9	26.1 ± 2.9	0.104
Follow-up, years	13.4 ± 1.5	13.1 ± 1.7	0.238

^α^ Data are presented as numbers or means ± standard deviations

UKA, unicompartmental knee arthroplasty; MOWHTO, medial open-wedge high tibial osteotomy; BMI, body mass index

The clinical outcomes significantly improved in both groups at the latest follow-up and there was no significant difference in any of the postoperative clinical outcomes including the proportion of MCID achievement between the two groups Table 20. Regarding radiologic assessment, the preoperative K–L grade progressed significantly more in the UKA group than in the MOWHTO group (*P* < 0.001). Moreover, the preoperative HKAA and MPTA had less varus alignment of lower limb and less tibia vara in the UKA group (HKAA, −5.0° ± 3.0°; MPTA, 86.6° ± 1.7°) than in the MOWHTO group (HKAA, −7.1° ± 3.0°; MPTA, 83.9° ± 1.9°; all for *P* < 0.001) (Table 3). Regarding the changes of patellofemoral joint, the incidence of radiologic progression of patellofemoral joint was significantly higher in the MOWHTO group (61.4%) than in the UKA group (43.1%; *P* = 0.013); however, the presence of anterior knee pain was not significantly different between the two groups at the latest follow-up (Table 4). Meanwhile, no significant difference in clinical outcomes or survivorship was found based on the subgroup analysis focusing on the overlapping indication for both operations (Table 5). Intraclass correlation (ICC) coefficients for all radiologic evaluations of intra- and inter-observer errors ranged from 0.84 to 0.94 and 0.86 to 0.96, respectively, showing excellent agreement [42].

**Table 2:** Comparison of clinical outcomes at preoperatively and the latest follow-up^α^

	UKA	MOWHTO	*P* value
ROM (FC), °			
Preoperative	4.7 ± 5.5	1.2 ± 2.6	< 0.001
Latest F/U	0.1 ± 0.6^†^	0.3 ± 1.2^†^	0.097
ROM (FF), °			
Preop	131.9 ± 11.8	132.4 ± 7.3	0.738
Latest F/U	134.8 ± 1.0^†^	134.7 ± 1.3^†^	0.482
AKS knee score			
Preoperative	60.1 ± 13.9	61.7 ± 6.1	0.357
Latest F/U	91.9 ± 8.1^†^	91.2 ± 7.2^†^	0.552
MCID achievement^‡^	71 (93.4)	134 (97.1)	0.456
AKS function score			
Preoperative	62.0 ± 10.2	63.7 ± 5.6	0.193
Latest F/U	93.2 ± 9.9^†^	93.6 ± 9.5^†^	0.775
MCID achievement^‡^	73 (96.1)	136 (98.6)	0.614
Total WOMAC score			
Preoperative	50.3 ± 12.1	48.2 ± 11.5	0.204
Latest F/U	10.5 ± 7.9^†^	12.3 ± 10.6^†^	0.258
MCID achievement^‡^	63 (82.9)	120 (87)	0.544
The presence of anterior knee pain			
Preoperative	N/A	N/A	N/A
Latest F/U	3 (3.9)	8 (5.8)	0.750

^α^ Data are presented as number (%) or means ± standard deviation

^†^ According to paired *t*-test, postoperative clinical outcomes were significantly improved after the procedure (*P* < 0.001)

^‡^The MCID value was set based on previous studies reporting 10 and 16.1 of total WOMAC score for UKA and MOWHTO, respectively; 7.2 and 6.4 of AKS knee score for UKA and MOWHTO, respectively; 9.7 and 5.9 of AKS function score for UKA and MOWHTO, respectively

*UKA* unicompartmental knee arthroplasty, *MOWHTO* medial open-wedge high tibial osteotomy, *ROM* range of motion, *FC* flexion contracture, *FF* further flexion, Preop. preoperative, *F/U* follow-up, *AKS* American knee society, *WOMAC* Western Ontario and McMaster Universities Osteoarthritis Index., *MCID* minimal clinically important difference, *N/A* not applicable

**Table 3:** Comparison of radiologic outcomes at preoperatively and the latest follow-up^α^

	UKA	MOWHTO	*P* value
Preop. KL grade of medial compartment 2 3 4	0 42 (53.2%) 37 (46.8%)	73 (52.1%) 50 (35.7%) 17 (12.2%)	< 0.001
HKAA, °			
Preoperative	−5.0 ± 3.0	−7.1 ± 3.0	< 0.001
Postoperative (6 weeks)	−1.6 ± 3.2	2.5 ± 2.8	< 0.001
Latest F/U	−3.5 ± 3.4	1.0 ± 3.1	< 0.001
MPTA, °			
Preoperative	86.6 ± 1.7	83.9 ± 1.9	< 0.001
Postoperative (6 weeks)	N/A	93.1 ± 3.2	N/A
Latest F/U	N/A	92.7 ± 3.2	N/A
mLDFA, °			
Preoperative	88.5 ± 2.0	88.9 ± 2.8	0.331
Postoperative (6 weeks)	N/A	88.5 ± 2.4	N/A
Latest F/U	N/A	88.5 ± 2.3	N/A

^α^ Data are presented as numbers or means ± standard deviation

UKA, unicompartmental knee arthroplasty; MOWHTO, medial open-wedge high tibial osteotomy; K–L, Kellgren-Lawrence; HKAA, hip-knee-ankle angle; MPTA, medial proximal tibial angle; mLDFA, mechanical lateral distal femoral angle; Preop., preoperative; Postop., postoperative; F/U, follow-up; N/A, not applicable

**Table 4:** Radiologic change of patellofemoral joint^α^

	UKA	HTO	*P* value
Preoperative K–L grade on Merchant view			0.194
Grade 0	7 (8.9)	22 (15.7)	
Grade 1	47 (59.5)	77 (55.0)	
Grade 2	25 (31.6)	37 (26.4)	
Grade 3	0 (0)	4 (2.9)	
Grade 4	0 (0)	0 (0)	
Postoperative K–L grade on Merchant view at the latest F/U			0.001
Grade 0	0 (0)	8 (6.1)	
Grade 1	33 (45.8)	30 (22.7)	
Grade 2	29 (40.3)	56 (42.4)	
Grade 3	10 (13.9)	38 (28.8)	
Grade 4	0 (0)	0 (0)	
Radiologic change of patellofemoral arthritis			0.013
Progression	31 (43.1)	81 (61.4)	
Nonprogression	41 (56.9)	51 (38.6)	

^α^ Data are presented as numbers (%) and values were analyzed in 219 knees preoperatively and in 204 knees with available radiographs postoperatively.

UKA, unicompartmental knee arthroplasty; MOWHTO, medial open-wedge high tibial osteotomy; K–L, Kellgren-Lawrence; F/U, follow-up

**Table 5:** Comparison of clinical outcomes at preoperatively and the latest follow-up in patients with the overlapping indication^α^

	UKA (*n* = 24)	MOWHTO (*n* = 21)	*P* value
ROM (FC), °			
Preoperative	3.1 ± 4.4	1.2 ± 2.7	0.078
Latest F/U	0.1 ± 0.1^†^	0.1 ± 0.1^†^	> 0.999
ROM (FF), °			
Preoperative	131.9 ± 12.1	132.9 ± 6.8	0.736
Latest F/U	135.0 ± 0.1^†^	134.8 ± 2.5^†^	0.666
AKS knee score			
Preoperative	64.7 ± 16.9	61.8 ± 5.5	0.468
Latest F/U	92.5 ± 6.9^†^	90.5 ± 7.5^†^	0.353
MCID achievement^‡^	24 (100)	21 (100)	> 0.999
AKS function score			
Preoperative	64.0 ± 10.2	64.5 ± 5.1	0.860
Latest F/U	93.9 ± 10.6^†^	88.6 ± 8.5^†^	0.070
MCID achievement^‡^	24 (100)	21 (100)	> 0.999
Total WOMAC score			
Preoperative	49.3 ± 10.1	47.2 ± 9.5	0.497
Latest F/U	9.7 ± 8.3^†^	11.5 ± 7.4^†^	0.258
MCID achievement^‡^	24 (100)	21 (100)	> 0.999
The presence of anterior knee pain			
Preoperative	N/A	N/A	N/A
Latest F/U	1 (4.2)	1 (4.8)	> 0.999
Survival			
TKA conversion	0	0	> 0.999
Reoperation	2 (8.3)	1 (4.8)	> 0.999

^α^ Data are presented as number (%) or means ± standard deviation

^†^ According to paired *t*-test, postoperative clinical outcomes were significantly improved after the procedure (*P* < 0.001)

^‡^The MCID value was set based on previous studies reporting 10 and 16.1 of total WOMAC score for UKA and MOWHTO, respectively; 7.2 and 6.4 of AKS knee score for UKA and MOWHTO, respectively; 9.7 and 5.9 of AKS function score for UKA and MOWHTO, respectively

UKA, unicompartmental knee arthroplasty; MOWHTO, medial open-wedge high tibial osteotomy; ROM, range of motion; FC, flexion contracture; FF, further flexion; Preop., preoperative; F/U, follow-up; AKS, American knee society; WOMAC, Western Ontario and McMaster Universities Osteoarthritis Index.; MCID, minimal clinically important difference; N/A, not applicable; TKA, total knee arthroplasty

**Fig. 2:**
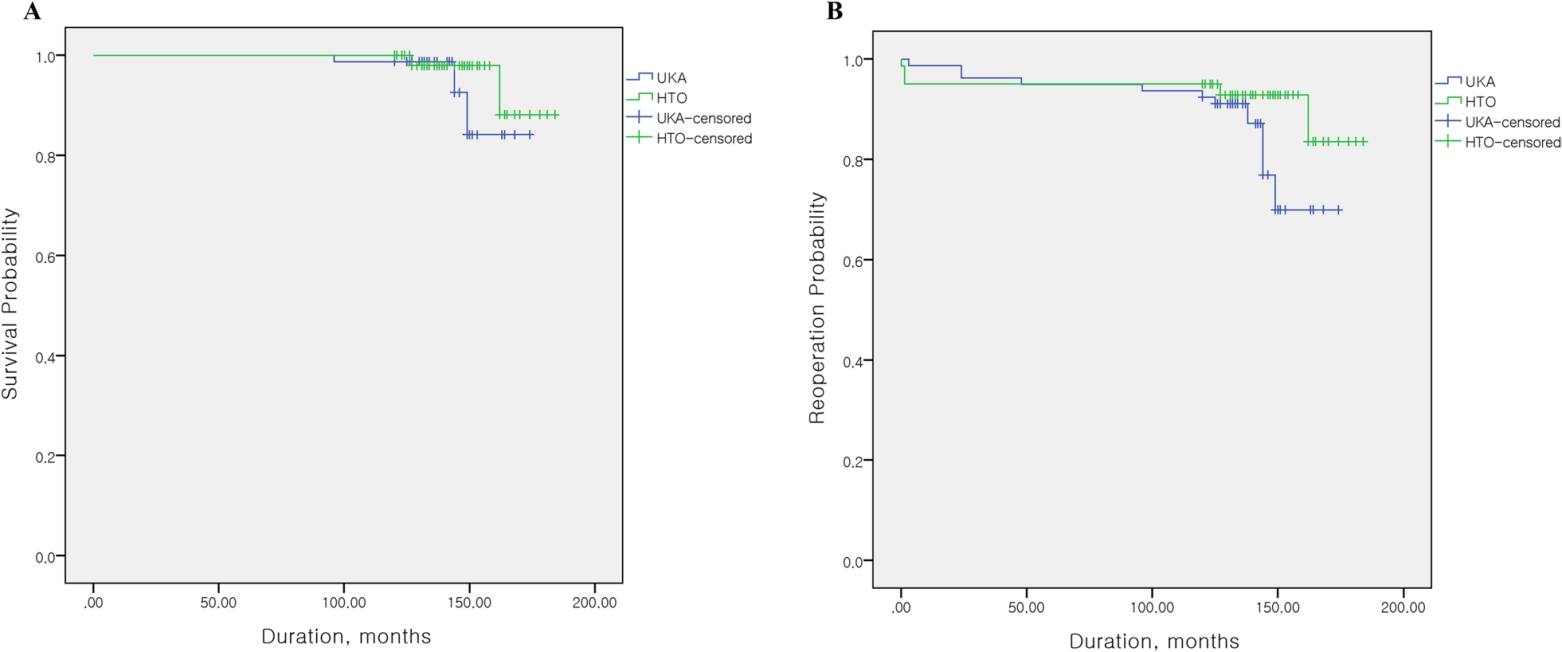
Kaplan–Meier survival analysis of conversion to total knee arthroplasty (A) and all-cause for reoperation (B) the UKA and MOWHTO groups at the minimum 10 years follow-up. UKA, unicompartmental knee arthroplasty; MOWHTO, medial open-wedge high tibial osteotomy

The survival rate at an average follow-up of 13 years was not significantly different between the two groups (UKA group versus MOWHTO group: 96.2% versus 98.6%, *P* = 0.257) and the reoperation rate was not also significantly different between the two groups (UKA group versus MOWHTO group: 86.1% versus 93.6%, *P* = 0.119) (Fig. 2). Two knees in the MOWHTO group and three knees in the UKA group were converted to TKA (Table 6). Postoperative complications occurred in seven cases in the UKA group and seven cases in the MOWHTO group (Table 6). However, the incidence of postoperative complications was not significantly different between the two groups (*P* = 0.550).

**Table 6:** Reasons for conversion to TKA or reoperation

	UKA		HTO	
Mode of failure	No	Time point	No	Time point
TKA conversion	3		2	
Progression of osteoarthritis	0		2	PO 7 and 13 years
Femoral component loosening	1	PO 12 years	N/A	
Tibial component loosening	2	PO 8 and 13 years	N/A	
Reoperation	7		7	
SSI (ID and PE exchange)	1	PO 1 year	N/A	
PE dislocation (PE exchange)	2	PO 2 and 4 years	N/A	
PE wearing (PE exchange)	4	PO 2, 8, 10, 10 years	N/A	
Wound dehiscence	N/A		5	All < PO 3 months
Fixation failure	N/A		2	PO 6 and 8 weeks

^α^ Data are presented as numbers

TKA, total knee arthroplasty; UKA, unicompartmental knee arthroplasty; HTO, high tibial osteotomy; PO, postoperative; N/A, not applicable; SSI, surgical site infection; ID, incision and drainage; PE, polyethylene

## Discussion

The principal finding of the current study was that both UKA and MOWHTO, when performed under their established indications [6, 7, 10–15] reported in the literature, demonstrated successful long-term clinical outcomes and survivorship, without significant difference in complication rates.

There is a lack of study comparing long-term results between UKA and MOWHTO. Recently, two studies performed long-term comparisons between the two surgeries and showed no significant difference in clinical outcomes and survival rates at the 10-year follow-up [2, 3]. Nevertheless, they compared long-term outcomes within a restricted cohort, where there was an overlap in the indication for both methods [2, 3]. This approach would have some limitation in its ability to determine the most optimal indication for each method [2, 3]. Therefore, the current study conducted a retrospective cohort comparison of UKA and MOWHTO with a minimum of 10 years of follow-up, including consecutive patients treated according to established indications [6, 7, 10–15] at a single institution. Although anatomical criteria for surgical selection, such as intra- versus extra-articular varus deformity, are well established, their impact on long-term survivorship remains controversial [43, 44]. This study demonstrated that when these indications were performed under their established indications [6, 7, 10–15] reported in the literature, both procedures resulted in excellent clinical outcomes with a high rate of MCID achievement sustained over more than 10 years. Like previous studies [2, 3], no significant differences were found in clinical outcomes and survival rates between UKA and MOWHTO. Furthermore, UKA and MOWHTO in the current study demonstrated excellent 10-year survival rates of 96.2% and 98.6%, respectively. Previous studies have reported comparable or slightly lower survival rates (UKA, 87.1%–96.1%; HTO, 87.7%–91.0%), which may be attributable to differences in patient selection, surgical technique and era, and fixation methods [2, 3, 45].

Interestingly, none of the patients who underwent UKA and MOWHTO converted to TKA owing to OA progression in the lateral or patellofemoral compartments over a 10-year follow-up. Only two patients (1.4%) with MOWHTO underwent conversion to TKA due to OA progression in the medial compartment. This is consistent with a previous study showing that OA progression in the lateral and patellofemoral compartments was not a significant concern for either method [2]. Although the current study showed that the incidence of OA progression in the patellofemoral compartment was significantly higher in MOWHTO than in UKA, the clinical outcomes, including the presence of anterior knee pain and survival, were similar between the two methods. This is consistent with a recent study reporting that arthritic changes in the patellofemoral joint did not affect long-term clinical outcomes or survivorship after MOWHTO, although degeneration of the patellofemoral joint was observed over time after MOWHTO [46]. Degeneration of patellofemoral joint might be associated with the natural progression of OA that occurs with aging, potentially leading to spontaneous deterioration in the stage of patellofemoral OA [46].

On the basis of the favorable long-term outcomes observed in our study, the importance of appropriate selection for UKA and MOWHTO should be emphasized. While these procedures are performed under their established indications [6, 7, 10–16] reported in the literature applied to well-selected patients, our results suggest that both can yield successful outcomes. Surgical decision-making is largely guided by the location of the tibial deformity (intra-articular versus extra-articular), degree of varus alignment, severity of arthritis, and patient characteristics such as age and activity level [1, 4, 5]. In our cohort, UKA was generally chosen for older patients (mean age 60.6 ± 5.1 years) with intra-articular varus deformity, advanced stage of arthritis, and relatively preserved alignment (mean preoperative MPTA: 86.6° ± 1.7°). In contrast, MOWHTO was preferred for younger patients (mean age 54.4 ± 8.6 years) with extra-articular proximal tibial deformity, less severe arthritis, and lower MPTA values (83.9° ± 1.9°), where angular correction was required. A recent study demonstrated that tibial varus deformity differentially affected clinical outcomes after MOWHTO and UKA, which aligned with the current study [47]. Moreover, Mullaji et al. [44] reported that knees with more than 10° of varus deformity due to extra-articular tibial bowing are at greater risk of postoperative malalignment after UKA, potentially compromising implant longevity. This supports our institutional practice of applying a conservative threshold of 10° varus for UKA candidacy [3, 48, 49]. MOWHTO, by contrast, allows for controlled correction of alignment and is more suitable in such deformities. These considerations are reflected in the surgical choices made in our cohort and help to explain the favorable long-term results observed when procedures are performed according to their respective indications.

Despite its informative findings, this study has several limitations. First, the retrospective design inherently introduces potential selection bias and confounding, as treatment allocation was based on preoperative characteristics and surgeon preference. Nonetheless, the relatively high follow-up rate (82.6%) over a minimum of 10 years is a notable strength. Second, significant differences in baseline characteristics—such as age, osteoarthritis severity, and limb alignment—between the two groups may have influenced outcomes, despite comparative analyses. In the UKA cohort, the inclusion of both fixed- and mobile-bearing implants may have introduced bias, although previous studies suggest comparable long-term outcomes between the two designs [50, 51]. Third, owing to the retrospective nature of the study, data regarding return to sports, recreational activity, and forgotten joint scores were not available. Additionally, sex-related disparities were observed, though female predominance is well-documented in East Asian populations undergoing HTO or knee arthroplasty [2]. Subgroup analysis for patients with overlapping indications was performed, but the small sample size resulted in insufficient statistical power for significant comparison. Furthermore, deformity type (intra-articular versus extra-articular) was not explicitly classified, although differences in MPTA and alignment indirectly reflect this distinction. Lastly, although radiographic progression of patellofemoral arthritis was evaluated, only the presence of anterior knee pain was recorded, and no patellofemoral-specific clinical scoring system was used, which may have led to underestimation of its clinical relevance.

## Conclusions

Both UKA and MOWHTO demonstrated satisfactory long-term outcomes for the treatment of medial compartmental OA when performed under their established indications [6, 7, 10–15] reported in the literature. In our cohort, patients undergoing UKA were older, had more advanced OA, demonstrated less varus alignment, and showed a larger MPTA compared with those treated with MOWHTO, consistent with previously described selection criteria. Radiographic progression of patellofemoral arthritis occurred more frequently after MOWHTO than after UKA, although this was not associated with clinical significance.

## Data Availability

Data and materials are available when requested to corresponding author. The manuscript is original research that has not been published and is not under consideration elsewhere. All of the authors participated in the preparation of the manuscript. All authors had access to the data and a role in writing the manuscript.

## Abbreviations

OA: Osteoarthritis
UKA: Unicompartmental knee arthroplasty
MOWHTO: Medial open-wedge high tibial osteotomy
TKA: Total knee arthroplasty
K–L: Kellgren-Lawrence
BMI: Body mass index
MPTA: Medial proximal tibial angle
ROM: Range of motion
FC: Flexion contracture
FF: Further-flexion
AKS: American-Knee-Society
WOMAC: Western-Ontario and McMaster-Universities-Osteoarthritis-Index
MCID: Minimal clinically important difference
AP: Anteroposterior
HKAA: Hip-knee-ankle angle
mLDFA: Mechanical lateral distal femoral angle
ICC: Intraclass correlation
